# Isoliquiritigen Enhances the Antitumour Activity and Decreases the Genotoxic Effect of Cyclophosphamide

**DOI:** 10.3390/molecules18088786

**Published:** 2013-07-24

**Authors:** Hong Zhao, Xuan Yuan, Defang Li, Hongmei Chen, Jiangtao Jiang, Zhiping Wang, Xiling Sun, Qiusheng Zheng

**Affiliations:** 1 School of Pharmacy, Shihezi University, Shihezi 832002, China; 2 Lanzhou University Second Hospital, Lanzhou University, Lanzhou 730000, China; 3 School of integrated traditional Chinese and Western Medicine, Binzhou Medical College, Yantai 264000, China; 4 Life Science School, Yantai University, Yantai 264000, China

**Keywords:** isoliquiritigenin, antitumour, cyclophosphamide, genotoxic effect

## Abstract

The aim of this study was to evaluate the antitumour activities and genotoxic effects of isoliquiritigenin (ISL) combined with cyclophosphamide (CP) *in vitro* and *in vivo*. U14 cells were treated with either of ISL (5–25 μg/mL) or CP (0.25–1.25 mg/mL) alone or with combination of ISL (5–25 μg/mL) and CP (1.0 mg/mL) for 48 h. The proliferation inhibitory effect *in vitro* was evaluated by MTT and colony formation assays. KM mice bearing U14 mouse cervical cancer cells were used to estimate the antitumour activity *in vivo*. The genotoxic activity in bone marrow polychromatic erythrocytes was assayed by frequency of micronuclei. The DNA damage in peripheral white blood cells was assayed by single cell gel electrophoresis. The results showed that ISL enhanced antitumour activity of CP *in vitro* and *in vivo*, and decreased the micronucleus formation in polychromatic erythrocytes and DNA strand breaks in white blood cells in a dose-dependent way.

## 1. Introduction

Cancer is one of the major causes of mortality in humans throughout the World. According to a report dealing with the incidence and mortality of cancer in the World, about 12.7 million cancer cases and 7.6 million cancer deaths were estimated to have occurred in 2008; of these, 56% of the cases and 64% of the deaths occurred in the economically developing world [1]. In China, cancer has become the leading cause of deaths among urban and rural residents. The high rate of malignancy of these neoplasms is associated with rapid proliferation, numerous early metastases and high resistance to conventional treatments involving surgery, chemotherapy and radiation. Although preventive measures like chemotherapy are very useful, these often result in manifestation of chronic side effects [2,3]. Cyclophosphamide (CP), also known as cytophosphane, is a nitrogen mustard alkylating agent. It has been used to treat leukemia [4], breast cancer [5], small cell lung cancer [6], cervical cancer [7], non-Hodgkin’s lymphoma [8] and so on. However, it has severe and life-threatening adverse effects. Some reports indicated that CP could cause temporary or (more rarely) permanent sterility [9] and produce chromosome damage [10], micronuclei [11], sister chromatid exchanges and DNA strand breaks in many kinds of mouse cells [12]. Therefore, it is desirable to find a compound that can decrease the genotoxic effects of cyclophosphamide without having any negative effect on its antitumour activity.

**Figure 1:**
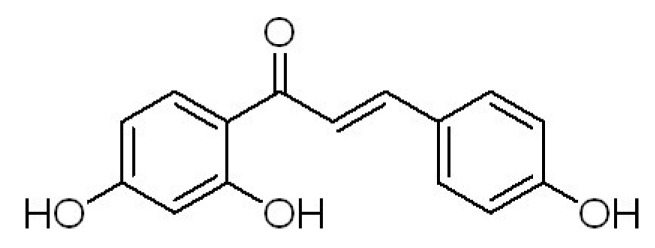
The chemical structure of ISL.

Licorice (*Glycyrrhiza uralensis*) has been used for more than four millennia as a flavoring agent in foods, beverages, and tobacco, and to treat individuals with gastric or duodenal ulcers [13], sore throats, coughs, bronchitis, arthritis [14], and allergies [15]. Moreover, many studies have revealed that several licorice derived compounds, *i.e.*, glycyrrhizin, isoliquiritigenin (ISL, Figure 1), licochalcone, and glabridin, have a variety of pharmaceutical effects [16,17,18,19]. In addition, ISL, a dietary flavonoid, has been evaluated in terms of its antioxidative effects [20], antiplatelet aggregation effects [21], and estrogenic properties [22]. In particular, ISL can suppress proliferation and induce apoptosis in many kinds of cancer cells, including human promyelocytic cell line [23], human glioma cells [24], human uterine leiomyoma cells [25], colon cancer cells [26], human prostate cancer [27], and human hepatoma cells [28]. ISL also suppresses migration and invasion in human breast cancer cells [29] and mouse renal cell carcinoma [30]. The results from our lab showed that ISL was able to induce monocytic differentiation in HL-60 human leukemia cells [31,32] or B16F0 mouse melanoma cells [33], suppress proliferation and induce apoptosis in HeLa human cervical carcinoma cells [34,35] and SKOV-3 human ovarian carcinoma cells [36], we also found that ISL and CP have synergistic action in inhibition of proliferation of U14 cells [37]. However, useful information about the pharmacological and genotoxic effects of ISL combined with chemotherapeutic agents is scarce. In this study, the effects of ISL on the antitumour acitivity and genotoxic effect of CP were investigated.

## 2. Results and Discussion

### 2.1. High Concentration of ISL Significantly Enhances CP-Induced Inhibition of U14 Cells Proliferation *in Vitro*

**Figure 2:**
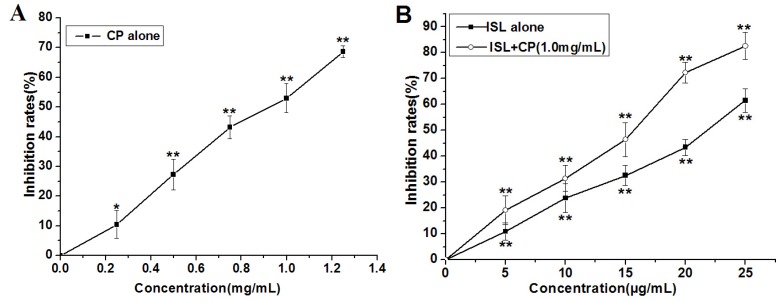
Effects of ISL, CP alone or their combination (ISL + CP) on U14 cells proliferation. (**A**) The inhibition rate on cell proliferation after 48 h treatment with CP (0, 0.25, 0.5, 0.75, 1.0, 1.25 mg/mL) alone; (**B**) The inhibition rate on cell proliferation after 48 h treatment with ISL (5, 10, 15, 20 and 25 μg/mL) alone or ISL (5, 10, 15, 20 and 25 μg/mL) co-treatment with CP (1.0 mg/mL). Data are presented as mean ± S.D. from three independent experiments. ** *p* < 0.01; * *p* < 0.05 *versus* control group.

**Figure 3:**
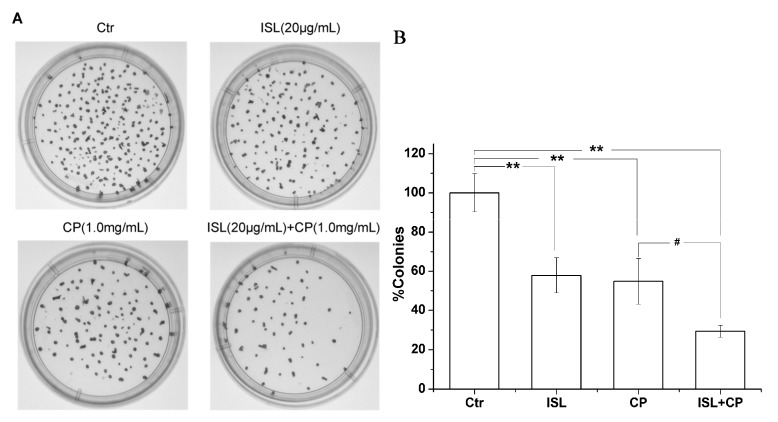
Effects of ISL, CP alone or their combination (ISL + CP) on the clonogenic potential in U14 cells. U14 cells were treated with ISL (20 μg/mL) or CP (1.0 mg/mL) alone or with their combination and allowed to proliferate for eight days. (**A**) Representative images of colony forming assays. (**B**) Colonies were counted and expressed as a percent of the control. Data are presented as mean ± S.D. from three independent experiments. ** *p* < 0.01 *versus* control group; *^#^ p* < 0.05 *versus* CP-treated group.

As shown in Figure 2, after treatment by CP (0, 0.25, 0.5, 0.75, 1.0, 1.25 mg/mL) or ISL (5, 10, 15, 20 and 25 μg/mL), respectively, for 48 h, a significant concentration-dependent reduction in cell viability was observed, the proliferation rate of 1.25 mg/mL CP-treated U14 cells decreased by 68.68%, 25 μg/mL ISL decreased by 61.57%. In view of the significant proliferation inhibition of U14 cells induced by CP, we chose the concentration of 1.0 mg/mL for the subsequent *in vitro* assays. The inhibition rate of co-treatment with 1.0 mg/mL CP and low concentration of ISL (5, 10, 15 μg/mL) is lower than that of 1.0 mg/mL CP alone, however the combination of ISL (20 μg/mL) significantly enhances CP-induced inhibition of U14 cells proliferation, the percentage of viable cells decreased from 47.00% (after treatment with CP 1.0 mg/mL) to 27.64% (treatment with the combination of 1.0 mg/mL CP and 20 μg/mL ISL). To further confirm the potentiation of co-treatment, the optimal combination of ISL 20 μg/mL and CP 1.0 mg/mL was used to investigate the effect on suppression of long-term colony formation. Exposure of U14 with ISL (20 μg/mL) and CP (1.0 mg/mL) resulted in a greater inhibition of colony formation than each agent alone (Figure 3), the colony-forming rate of cells exposed to ISL and CP was decreased by 70.64% compared with the control and 25.41% compared with CP alone.

### 2.2. Co-Treatment with ISL and CP Synergestically Decreases the Tumour Growth *in Vivo*

**Figure 4:**
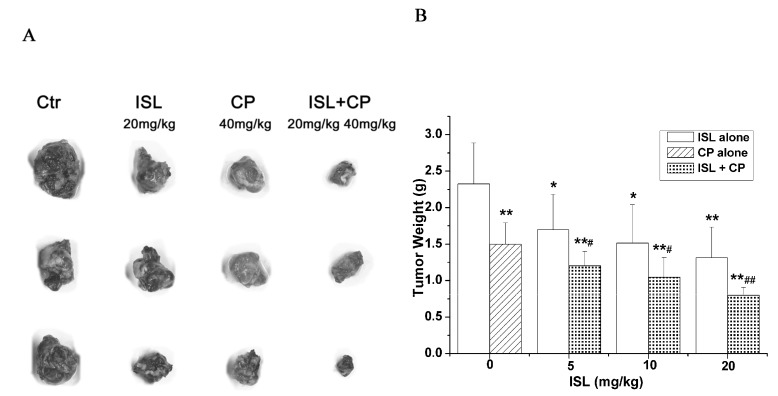
Synergestic inhibition of ISL and CP on tumour growth in *vivo*. KM mice bearing cervical cancer U14 cells were administrated with ISL (5~20 mg/kg, orally administered once a day for 10 consecutive days) or CP (40 mg/kg, injected by intraperitoneally a single dose of 40 mg/kg body weight at the first day). The implanted sarcomas were separated and weighed on day 11. (**A**) Images of the tumour morphology. (**B**) The quantitative results of tumor growth-inhibition. Data are presented as mean ± S.D. from 10 individual treatments. * *p* < 0.05; ** *p* < 0.01 *versus* control group; ^#^ *p* < 0.05; ^##^ *p* < 0.01 *versus* CP-treated group.

To further explore the possible synergistic effects of ISL with conventional chemotherapeutic drug, CP, mouse cervical cancer U14 cells were transplanted subcutaneously into KM mice. As shown in Figure 4, co-treatment with ISL (20 mg/kg) and CP (40 mg/kg) significantly inhibited the growth of cervical cancer U14 cells, the inhibition rate reached 65.66%, while the inhibition rate was 43.55% and 35.57% when treated with ISL(20 mg/kg) or CP (40 mg/kg) alone respectively (Figure 4).

### 2.3. ISL Inhibits the Micronuclei Yield Induced by CP

**Figure 5:**
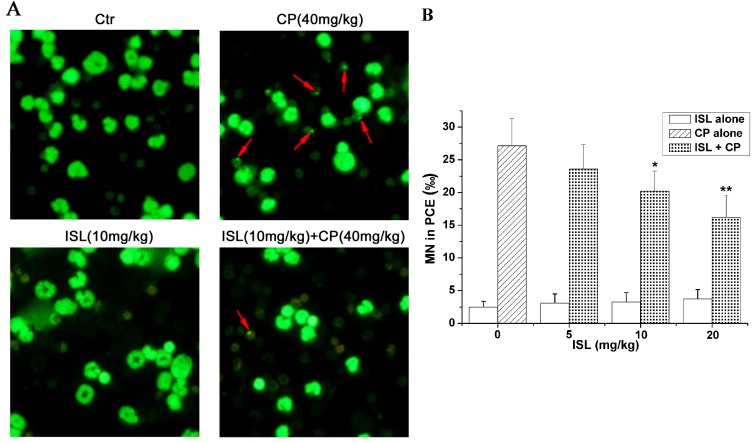
Pretreatment with ISL inhibits CP-induced micronucleus formation. Mice were pretreated with ISL (5, 10, 20 mg/kg) before CP treatment for three consecutive days. After CP (a single dose of 40 mg/Kg) treatment, mice were sacrificed after 24 h and the femoral bone marrow cells were collected. Smear slides of bone marrow cells were stained with AO. (**A**) Representative images of micronucleus formation assays. (**B**) The micronuclei (MN) in 1,000 polychromatic erythrocytes (PCEs) were counted under a fluorescence Carl Zeiss microscope. Data are presented as mean ± S.D. from 10 individual treatments; * *p* < 0.05, ** *p* < 0.01 *versus* CP-treated group.

CP alone significantly increases micronucleus formation in polychromatic erythrocytes (Figure 5), approximately 11 times higher than that of control group, while ISL alone has no influence on micronuclei. Pretreatment with ISL partially blocked CP-induced micronuclei in a dose-dependent way. The inhibition rate of ISL (20 mg/kg) treatment on micronuclei reached 41.4% (Figure 5).

### 2.4. ISL Inhibits the DNA Damage Induced by CP

**Figure 6:**
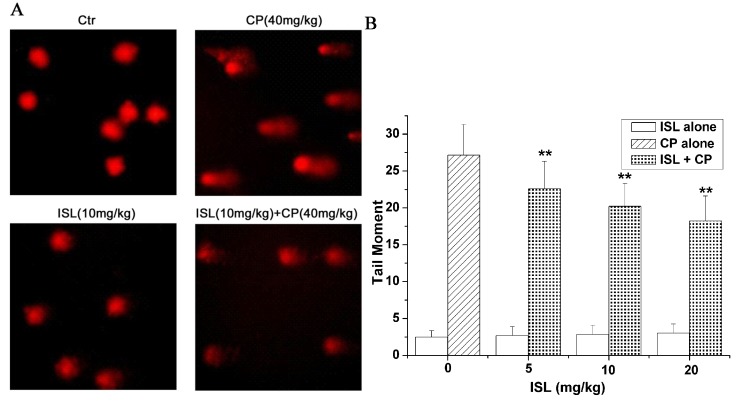
Pretreatment with ISL inhibits CP-induced DNA-damage. Mice were pretreated with ISL (5, 10, 20 mg/Kg) before CP treatment for 3 consecutive days. After CP (a single dose of 40 mg/Kg) treatment for 24 h, blood was obtained from murine tail tips. Comet assay was performed as described in Materials and Methods. (**A**) Representative images of Comet assay. (**B**) The tail moment was measured by CASP software. The mean value of the tail moment in a particular sample was taken as an index of DNA damage in this sample. Data are presented as mean ± S.D. from 10 individual treatments. * *p* < 0.05, ** *p* < 0.01 *versus* CP-treated group.

As shown in Figure 6, CP alone significantly increased the DNA damage detected by SCGE, as shown by the significant increase of olive tail moment; ISL alone had no influence on DNA, and when co-treated with combination of ISL and CP, the olive tail moment decreased in a dose-dependent manner (Figure 6).

### 2.5. Discussion

Improvements in the therapy of malignant disease during the past two decades have allowed long-term survival and cure in many patients. However, in recent years secondary malignancies have been increasingly recognized as an important late complication after chemo- and radiotherapy [38,39]. Some reports indicate that a few secondary malignant cases are related to cyclophosphamide (CP) [40,41,42] and that CP could induce the formation of urinary bladder tumours in rats [43], while still being used extensively for its efficacy on primary tumours, as the first-line chemotherapy agent against breast cancer, small cell lung cancer, cervical cancer and non-Hodgkin’s lymphoma. It has been shown that CP could produce chromosome damage, micronuclei, sister chromatid exchanges and DNA strand breaks in many kinds of mouse cells [10,44,45]. It is obvious that all above indices are related with carcinogenesis. Therefore, it is necessary to find a compound that can decrease the genotoxic effects of CP without having any negative effect on its antitumour activity.

ISL, a dietary flavonoid, exhibits antitumor activities, including the capability to suppresses proliferation and induce apoptosis in human cervical carcinoma cells [35], to suppresses migration and invasion of human breast cancer cells [29], and to induce monocytic differentiation in HL-60 cells [31]. Previous studies indicated that flavonoids may protect the cells from toxicity induced by CP [46]. ISL has been found to counteract the side effects of cisplatin therapy in cancer patients [47] or to reduce pulmonary metastasis, without any weight loss or leukocytopenia [30]. However, limited information is available concerning the antitumour activity and genotoxic effects of ISL combined with CP. In this study, the antitumour activity and genotoxic effect of ISL combined with intraperitoneal injection of cyclophosphamide was investigated. A novel finding obtained from this study is that a combination of ISL and CP significantly inhibited the U14 tumour growth *in vitro* and *in vivo*, and the inhibition ratio reached 82.61% and 65.66% respectively. More important, ISL partially decreased CP-induced micronuclei formation, and dose-dependently (5, 10, 20 mg/kg) inhibited CP-induced DNA strand breaks, making ISL may be a candidate to decrease the genotoxic effects of chemotherapy with CP.

## 3. Experimental

### 3.1. Materials

Isoliquiritigenin (ISL, purity ≥ 98%) was purchased from Jiangxi Herb Tiangong Technology Co., Ltd. (Nanchang, China). Cyclophosphamide (CP) was purchased from Jiangsu Hengrui Medicine Co., Ltd. (Lianyungang, China). Ethidium bromide, acridine orange and Triton X-100 were purchased from Sigma Chemical Co. (St. Louis, MO, USA). Low melting point (LMP) agarose and normal agarose (electrophoresis grade) were obtained from Gibco-BRL (Grand Island, NY, USA). Heparin sodium was bought from Roche (Sao Paulo, SP, Brazil) under the commercial name Liquemine.

### 3.2. Animal Preparation

SPF KM female mice, aged 5–7 weeks and weighed from 18 to 22 g, were obtained from the Institute of Laboratory Animal Science, Chinese Academy of Medical Sciences, Beijing, China. The mice were acclimatized to laboratory conditions (22 ± 3 °C and 60% humidity) for 7 days, with a commercial standard mouse cube diet (Shihezi University Laboratory Animal Center, Xinjiang, China) and water *ad libitum*. After acclimatization, the mice were randomly divided into control and treated groups.

### 3.3. Cell Culture

Mouse cervical cancer U14 cells were obtained from China Center for Type Culture Collection (CCTCC), Wuhan, China. The cells were maintained in RPMI 1640 supplemented with 10% FBS, 100 U/mL penicillin, and 100 μg/mL streptomycin at 37 °C with 5% CO_2_. Cells were split every three days and were diluted one day before each experiment.

### 3.4. Cell Viability Assay

Cell viability was measured via 3-(4,5-dimethylthiazol-2-yl)-2,5-diphenyltetrazolium bromide (MTT) assay [48]. Cells were washed with fresh media, cultured in 96-well plates (Nunc, Roskilde, Denmark) at 1 × 10^5^ cells/mL, and then incubated with CP (0, 0.25, 0.5, 0.75, 1.0, 1.25 mg/mL) or ISL (0, 5, 10, 15, 20 and 25 μg/mL) or their combination for 48 h. The medium was aspirated after incubation, and then fresh medium containing 10 μL of 5 mg/mL MTT was added. The medium was removed after 4 h and replaced with blue formazan crystal dissolved in 150 μL DMSO. Absorbance at 490 nm was measured using a fluorescent plate reader (Millipore Bedford, MA, USA). Data were expressed as the percentage of cell viability compared with the control (DMSO). The inhibition rate was quantified using the following formula:

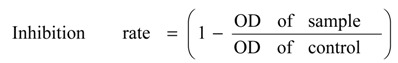


### 3.5. Colony Formation Assay

For this assay, cells were seeded at 500 cells per 100 mm culture dish and allowed to attach overnight [49]. The cells were treated with ISL (20 μg/mL), CP (1.0 mg/mL) or their combination and maintained under standard cell culture conditions at 37 °C and 5% CO_2_ in a humid environment. After 8 days, the dishes were washed twice in PBS, fixed with methanol, stained with Giemsa dye (Sigma), washed with PBS and air dried. Colonies exhibiting a minimum of 50 viable cells were counted. Colony plating efficiency was calculated to be the number of viable plated cells, and was expressed as a percentage of inoculated cells. The percent of colonies was calculated using the number of colonies formed in treatment divided by number of colonies formed in control group.

### 3.6. *In Vivo* Antitumour Activity Assay

KM mice were implanted subcutaneously with 0.2 mL (1 × 10^7^ mL in sodium chloride) U14 cells. After implantation for 24 h, mice bearing U14 were randomly assigned to eight groups (each group consisted of 10 mice) as follows: (1) control, (2) 40 mg/kg CP alone, (3) 5 mg/kg ISL, (4) 10 mg/kg ISL, (5) 20 mg/kg ISL, (6) 5 mg/kg ISL combined with 40 mg/kg CP, (7) 10 mg/kg ISL combined with 40 mg/kg CP and (8) 20 mg/kg ISL combined with 40 mg/kg CP. ISL, suspended in 0.5% carboxymethyl cellulose sodium (CMC-Na), was orally administered once a day for 10 consecutive days. CP, dissolved in saline, was injected intraperitoneally as a single dose of 40 mg/kg body weight at the first day. Controls received the vehicle alone (20 mL/kg). Mice were sacrificed by cervical dislocation on day 11. Implanted sarcomas were separated and weighed, then the tumor inhibition rate (TIR) was calculated according to the following formulate: TIR (%) = (WC − WE)/WE × 100%. WC: Mean tumor weight in Group 1 (control group); WE: Mean tumor weight in Group 2–8 (tested groups) respectively; >30% was regarded as having inhibitory effect.

### 3.7. Micronucleus Formation Assay

After acclimation, eighty female mice were randomly assigned to the eight groups as above mentioned. ISL was administered orally for three consecutive days. After the last administration, a single dose of CP 40 mg/kg body weight was injected intraperitoneally while the vehicle alone (20 mL/kg) was injected as the control. Blood (about 50 μL) was obtained 24 h after CP treatment from murine tail tips by a small incision and immediately mixed with heparin sodium (20 μL). The animals were then sacrificed by cervical dislocation. Bone marrow smears and staining were done following the procedure described by Tinwell [50]. Femoral bone marrow was flushed out by using 1% sodium citrate solution at 37 °C. The marrow was homogenized and centrifuged at 1,000 *g* for 5 min. The supernatant was decanted and the pellet was homogenized with the residual fluid and add 2 mL PBS buffer. After harvesting and washing, cells were stained with 50 µg/mL acridine orange and visualized with a Carl Zeiss fluorescence microscope with the corresponding filters (470–490 nm excitation and 515 nm emission wavelengths). For each animal, 1,000 polychromatic erythrocytes were examined by utilizing a number of fields, and the number of micronucleated polychromatic erythrocytes was recorded. Every individual experiment was performed in triplicate.

### 3.8. Comet Assay

The alkaline version of the comet assay (single cell gel electrophoresis, SCGE) was performed according to the protocol developed by Wang *et al.* [51]. Blood cells/heparin mixtures (7 µL) were embedded in LMP agarose (93 µL, 0.75 g/100 mL PBS) and the resulting mixture was spread over a precoated microscope slide (1.5 g/100 mL agarose). A cover glass was then gently placed over the slide and put at 4 °C for 5 min to allow gel solidification. After removal of the coverslips, the cells were lysed at 4 °C for at least one h in a freshly prepared, ice-cold solution of 2.5 M NaCl, 100 mM Na2EDTA, 10 mM Trizma Base, 1% Na-lauryl sarcosinate, pH 10, and 1% Triton X-100, and 10% fresh DMSO added. Shortly after lysis, the slides were placed on a horizontal electrophoresis unit. Then they were exposed to alkali (300 mM NaOH, 1 mM Na2EDTA, pH ± 13) at 4 °C for 20 min, to allow DNA unwinding. Electrophoresis was performed at 300 mA and 25 V (0.90 V/cm) at 4 °C for 15 min. The slides were then neutralized (Tris 0.4 M, pH 7.5), stained with ethidium bromide (20 mg/mL), and were observed and photographed at 400× magnification using a Carl Zeiss reverse fluorescence microscope equipped with an excitation filter (BP 546/12 nm) and a barrier filter (590 nm). The DNA damage degree was assessed with CASP, a free SCGE analysis system published by Konca *et al.* [52]. The images of one hundred cells were randomly selected from each slide and the tail moment was measured. The tail moment is positively correlated with the level of DNA breakage in a cell. The mean value of the tail moment in a particular sample was taken as an index of DNA damage in this sample.

### 3.9. Statistical Analysis

The results were expressed as mean ± S.D. and analyzed by one-way analysis of variance (ANOVA). The values with *p* < 0.05 were considered as statistically significant. The analyses were carried out using the Origin 8.0 software (Origin Lab Corporation, Northampton, MA, USA).

## 4. Conclusions

In conclusion, our studies indicated that ISL significantly inhibits the genotoxic effects induced by CP, and enhances the antitumor activity of CP both *in vitro* and *in vivo*. Therefore, ISL, is a powerful candidate drug, to decrease the toxic effects and to enhance the therapeutic effects of the chemotherapy with cyclophosphamide.
